# Ly6C^low^ and Not Ly6C^high^ Macrophages Accumulate First in the Heart in a Model of Murine Pressure-Overload

**DOI:** 10.1371/journal.pone.0112710

**Published:** 2014-11-21

**Authors:** Christina Weisheit, Yunyang Zhang, Anton Faron, Odilia Köpke, Gunnar Weisheit, Arne Steinsträsser, Stilla Frede, Rainer Meyer, Olaf Boehm, Andreas Hoeft, Christian Kurts, Georg Baumgarten

**Affiliations:** 1 Department of Anesthesiology and Intensive Care Medicine, University Hospital Bonn, Bonn, Germany; 2 Geschwister-Scholl-Gymnasium, Academic High School Daun, Daun, Germany; 3 Institute of Physiology II, University of Bonn, Bonn, Germany; 4 Institute of Experimental Immunology, University Hospital Bonn, Bonn, Germany; Albert Einstein College of Medicine, United States of America

## Abstract

Cardiac tissue remodeling in the course of chronic left ventricular hypertrophy requires phagocytes which degrade cellular debris, initiate and maintain tissue inflammation and reorganization. The dynamics of phagocytes in left ventricular hypertrophy have not been systematically studied. Here, we characterized the temporal accumulation of leukocytes in the cardiac immune response by flow cytometry and fluorescence microscopy at day 3, 6 and 21 following transverse aortic constriction (TAC). Cardiac hypertrophy due to chronic pressure overload causes cardiac immune response and inflammation represented by an increase of immune cells at all three time points among which neutrophils reached their maximum at day 3 and macrophages at day 6. The cardiac macrophage population consisted of both Ly6C^low^ and Ly6C^high^ macrophages. Ly6C^low^ macrophages were more abundant peaking at day 6 in response to pressure overload. During the development of cardiac hypertrophy the expression pattern of adhesion molecules was investigated by qRT-PCR and flow cytometry. CD11b, CX3CR1 and ICAM-1 determined by qRT-PCR in whole cardiac tissue were up-regulated in response to pressure overload at day 3 and 6. CD11b and CX3CR1 were significantly increased by TAC on the surface of Ly6C^low^ but not on Ly6C^high^ macrophages. Furthermore, ICAM-1 was up-regulated on cardiac endothelial cells. In fluorescence microscopy Ly6C^low^ macrophages could be observed attached to the intra- and extra-vascular vessel-wall. Taken together, TAC induced the expression of adhesion molecules, which may explain the accumulation of Ly6C^low^ macrophages in the cardiac tissue, where these cells might contribute to cardiac inflammation and remodeling in response to pressure overload.

## Introduction

Cardiovascular diseases are among the leading causes of death worldwide. Left ventricular (LV) hypertrophy is a main determinant of morbidity and mortality as it occurs in response to various stimuli, including systemic arterial hypertension, aortic stenosis, or remodeling of the myocardium after myocardial infarction (MI) [1]. Despite underestimation of its deleterious consequences, arterial hypertension leads to chronic pressure overload of the LV and initiates a remodeling process which changes tissue morphology and function. At the present time therapeutic approaches to modulate cardiac remodeling are still limited and presume an early diagnosis and consequent therapy of the initial stimuli [2]. Heart failure is the end stage disease of many, initially protective neurohumoral and inflammatory compensatory mechanisms which become harmful but in the course of disease [3], [4].

These mentioned compensatory mechanisms are accompanied by the activation of the cardiac immune response [5]. As the heart comprises more than just cardiac myocytes, e.g. endothelial cells, fibroblasts and a diversity of resident immune cells it becomes more and more essential to decode the cells’ particular function and intercellular communication [6]. The complex pro- and anti-inflammatory response after MI has already been described as a monocyte-dependent process [7]. In addition to monocytes, neutrophils are rapidly recruited over the first 24 hours after MI to the site of injury and start degrading extracellular matrix components as well as phagocytising dead cells. According to several surface markers it is possible to distinguish between resident and inflammatory macrophages and to describe a two phasic monocyte/macrophage response after MI [8]. The first phase is mainly affected by Ly6C^high^, inflammatory monocytes/macrophages, while the second phase is dominated by the accumulation of Ly6C^low^, tissue repairing monocytes/macrophages [9].

Current findings of Epelman et al. highlight that the immune system in the heart is uniquely adapted to the demands of physiological and pathological stress in steady state and after injury [10]. However, the dynamics of the immune response in the context of chronic pressure overload and consecutive LV tissue remodeling are incompletely understood. Our group recently verified the impact of MAC2^+^ cells in the development of cardiac hypertrophy but has not yet characterized the distinct macrophage subpopulations according to their surface expression of F4/80 and Ly6C following the definition of Geissman et al. [8], [11]. Recently using a murine model of urinary tract infection we found that F4/80^+^, Ly6C^high^ phagocytes showed pro-inflammatory helper macrophage functions by secreting TNF, while F4/80^+^, Ly6C^low^ phagocytes acted as sentinel macrophages and displayed helper-cell functions by secreting CXCL2, allowing neutrophil transepithelial migration [12].

The process of recruitment and activation of innate and adaptive immune cells could provide the initial point for immune modulatory strategies to ameliorate LV remodelling and cardiac function. Recent studies highlight that uncontrolled, overshooting monocyte response can impair scar formation after myocardial infarction [13], [14]. Recruitment of monocytes and neutrophils from the circulatory system are important for the induction and maintenance of inflammatory processes. After myocardial infarction, recruited monocytes and macrophages play a critical role in cardiac tissue remodeling [15]. The migration of myeloid cells is controlled by specific chemoattractants, called chemokines, and the expression of adhesion molecules on the surface of immune cells and endothelial cells. Here, the expression of the integrin heterodimer CD11b, also known as Mac-1, on the surface of monocytes and granulocytes directly influences cell adhesion and transendothelial migration as it interacts with the intercellular adhesion molecule 1 (ICAM-1) on endothelial cells [16]–[18]. Moreover, platelet/endothelial-cell adhesion molecule 1 (PECAM1), also known as CD31 has been reported to be another important junctional molecule, for which leukocytes express ligands, that facilitates cell adhesion and extravasation [19], [20]. In that context, the chemokine receptor CX3CR1, which is also known as fractalkine receptor, contributes to cell recruitment during inflammation through chemotaxis and mediation of adhesion [21]–[23]. Moreover, CX3CR1 can be used to distinguish between different monocyte/macrophage populations, as Ly6C^low^ monocytes/macrophages express more CX3CR1 than Ly6C^high^ cells [24], [25]. In addition, CX3CR1 is required for monocyte crawling or patrolling in the lumen of the blood vessels [26]. In the kidney, the sensing of a Toll-like-receptor 7 dependent danger signals triggered the intravascular retention of Ly6C^low^ monocytes by the endothelium, requiring CX3CR1 expression by Ly6C^low^ monocytes and CD11b [27]. These results suggest that Ly6C^low^ monocytes behave as sentinels in the vessels, rarely extravasating in comparison to Ly6C^high^ monocytes following Listeria infections [26], surveying the integrity of the endothelium. Their impact in orchestrating immune responses under sterile conditions remained undescribed, yet [27].

Thus, we hypothesized that pressure overload attracts specific phagocytes necessary for remodelling the myocardium. Referring to the findings observed in response to MI we proposed temporally coordinated invasion of different types of monocytes. This may be explained by a dynamic expression of adhesion molecules on both partner endothelial cells as well as leukocytes.

## Materials and Methods

### Animal Handling and Care

Female C57BL/6 mice (20–22 g) between 8 and 12 weeks of age were purchased from Charles River or bred at the central animal facilities of the medical faculty of Bonn (HET, House of Experimental Therapy). CX3CR1^−/−^ mice were purchased by Jackson Laboratory and bred with C57BL/6 mice to receive heterozygous CX3CR1^+/GFP^ mice. All mice were maintained under specific pathogen–free condition in isolated, ventilated cages with free access to water and food.

### Ethics Statement

All animal experiments were performed in accordance with the Guide for the Care and Use of Laboratory Animals published the US National Institutes of Health (NIH Publication No. 85–23, revised 1996). Treatment protocols were approved by the district government of Northrhine Westphalia, here the “State Office for protection of nature, environment and consumers” (LANUV Landesamt für Natur, Umwelt und Verbraucherschutz, Leibnizstraße 10, 45659 Recklinghausen, Germany), which is the responsible agency. The approval number is: 84–02.04.2011.A313. Animal experiments and handling were also supervised by the central animal facilities of the medical faculty Bonn (HET, House of Experimental Therapy, Sigmund-Freud Str. 25, 53127 Bonn, Germany). All surgical interventions were performed under anesthesia and analgesia as described below, and all efforts were made to minimize suffering.

### Transverse aortic constriction (TAC)

The TAC model was used to generate pressure overload-induced heart failure. Mice were anaesthetized with isoflurane (2 vol%), intubated with a 22 G tube and ventilated 10 ml/kg BW, 120 breaths/min with a small animal ventilator, purchased from Harvard Apparatus (Holliston, MA). A 27 G needle was used to standardize the degree of aortic constriction. Sham control animals underwent intubation and surgery without aortic restriction. For analgesia, buprenorphine was administered (0.1 mg/kg subcutaneously) before surgical intervention started and 8h later. Mice were sacrificed 3, 6 and 21 days following surgical intervention.

### Determination of Heart-Weight/body-Weight-Index

The body weight of each mouse was measured before sacrificing the animals. Weight of the whole heart was determined after carefully removing the organ from the murine thorax and washing out the blood with PBS. The index was calculated in mg/g.

### Perfusion

For some experiments it was necessary to perfuse the circulation of the mice before harvesting the hearts. For this, a 23 G needle was placed in the right ventricle of the murine heart and the circulation was perfused with 37°C warm PBS lacking Ca2^+^ and Mg2^+^ for 3 min until the heart, lung and liver were pale.

### Flow cytometry

Single cell suspensions from the LV were generated by mincing the tissue and 45 min digestion with 1 mg/ml Collagenase II, 100 mg/ml Collagenase I, 500 U/ml Hyaluronidase and 50 U/ml DNase I (all enzymes were purchased by Sigma, St. Louis, MO) in RPMI at 37°C under mild stirring. Blood samples were collected from the abdominal aorta and coagulation was stopped imediatly by adding 0.5 M EDTA solution. Samples were next washed with red blood cell lysis buffer (eBioscience, San Diego, CA). Cells were incubated with CD16/CD32 (2.4G2, BD Bioscience, Franklin Lakes, NJ) antibody at 4°C for 5 min to block non-specific binding of immunoglobulin to the Fc receptors. Cells were stained in PBS lacking Ca2^+^ and Mg2^+^ but containing 0.1% FCS (PAA) and 0.5% sodium azide. Titrated amounts of the following labelled antibodies from eBiosciences (San Diego, CA), BD Biosciences (Franklin Lakes, NJ) and BioLegend (San Diego, CA) were used: CD45 (AFS98), F4/80 (BM-8), Gr1 (RB6-8C5), Ly6C (HK1.4), Ly6G (1A8), CD11b (M1/70), CD31 (MEC 13.3), MHCII/I-A/I-E (2G9), ICAM-1 (YN1/1.7.4) and corresponding isotype controls. Cells were incubated with the antibodies for 15 min at 4°C in the dark and were washed afterwards. The analysis of the mean fluorescence intensity (MFI) refers to the fluorescence intensity of each event in average and may be taken as an indicator for the density of an antigen on the investigated cells.

For intensified ICAM-1 detection surface staining was combined with intracellular staining. We incubated single cell suspension of the LV for 4 h with 1 µl/ml Golgi-Plug (BD Bioscience), incubated them with CD16/CD32 to block Fc receptors, stained for surface markers, fixed BD Cytofix/Cytoperm and permeabilized with BD-Perm/Wash-Buffer according to the manufacturer's protocol (BD Bioscience).

Absolute cell numbers were determined by adding fixed numbers of CaliBRITE APC-beads (BD Biosciences) before measurement as an internal reference. We performed flow-cytometry on a FACSCanto II, LSR II and on a Fortessa (BD Bioscience) and analyzed the data with Flow-Jo software (TreeStar, Ashland, OR). Gating was performed as follows: First all cells were determined via FSC-A and SSC-A, then single cells were defined via SSC-A and FSC-W. Further gating strategies are indicated in respective figure legends.

### Histology

Immunofluorescence microscopy in cryosections: After overnight fixation in PFA4%, hearts were embedded in Tissue-Tec (Sakura) and immediately frozen at −80°C. Frozen blocks were cut into 30 µm sections and mounted on poly-L-lysine-coated glass slides (Menzel, Braunschweig, Germany). We used the following antibodies from eBiosciences and Bio Legend: CD31 (MEC 13.3), F4/80 (BM-8) and Ly6G (1A8). Furthermore we utilized AF488 labeled rabbit anti-GFP antibody purchased by Life technologies (Carlsbad, CA) and costained the slices with a donkey anti-rabbit antibody in AF647 (Life technologies) to minimize backgound fluorescence. Slices were analysed by immune fluorescence confocal microscopes (LSM 510 Meta and LSM 780, Carl Zeiss Microscopy GmbH, Jena, Germany). Data was analyzed with Imaris-software (Bitplane AG Zurich, Switzerland).

### Real Time PCR

Left ventricular tissue samples were stored at −80°C until homogenization in RNeasy Lysis Buffer (QIAGEN, Hilden, Germany) containing 1% 2-β-mercaptoethanol (Sigma-Aldrich, St. Louis, MO) by drawing through a 21 G needle>10 times. Subsequently total RNA was purified using the RNeasy mini kit (QIAGEN), according to the manufacturer's instructions. CD11b, CX3CR1, ICAM-1 and 18s mRNA expression were quantified by mRNA RT-PCR using TaqMan PCR detection System (Life Technologies, Carlsbad, CA). Messenger-RNA concentration was quantified by absorbance spectroscopy (Nanodrop Products, Wilmington, DE). The 18S ribosomal RNA was amplified as an internal control. Amounts of specific cDNA were finally normalized to 18s using the Δct method, and results are depicted as 2^ΔΔct^ values, as described [28], [29].

### Statistical analysis

Results are expressed as mean ±SEM. Statistical tests performed included Student's t test and one-way ANOVA with Tukey's multiple-comparison test and were performed with Prism 4 (GraphPad Software, Inc. La Jolla, CA). P values of less than 0.05 were considered significant.

## Results

### TAC leads to cardiac inflammation

**Figure 1:**
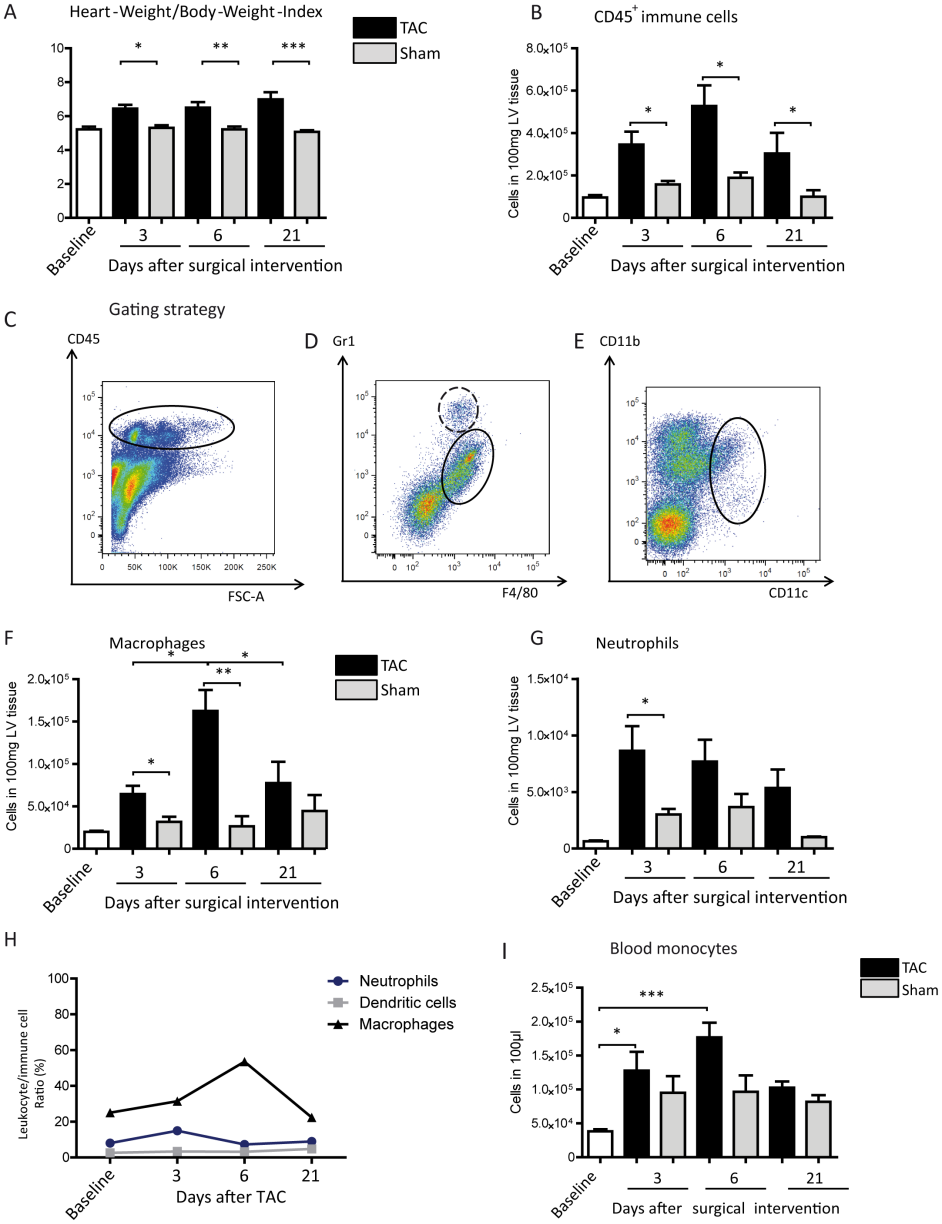
Morphological changes of the cardiac tissue in response to pressure overload. (A) Heart-Weight/Body-Weight-Index was calculated. Mean ±SEM, n: Baseline  = 12 mice, 3d TAC  = 11 mice, 3d Sham  = 10 mice, 6d TAC  = 12 mice, 6d Sham  = 12 mice, 21d TAC  = 10 mice, 21d Sham  = 10 mice. (B) The number of immune cells in cardiac tissue was determined by flow cytometry using CD45 surface staining. (C–E) Gating strategy of cardiac tissue; concatenated plots of 3 healthy baseline mice. (C) Cardiac immune cells (plot on the left, black ellipse) were determined via CD45 staining, previously gating all single cells. (D) Neutrophils (plot in the middle, black dashed circle) and macrophages (plot in the middle, black ellipse) were distinguished regarding the expression of Gr1 and F4/80 as indicated, previously gating all CD45^+^ cells. (E) Dendritic cells (plot on the right, black ellipse) were determined by CD11b and CD11c staining, previously gating all CD45^+^ cells. (F, G) The number of macrophages and neutrophils was determined by flow cytometry analysis. (B, F, G) Mean ±SEM; n: Baseline  = 10 mice, 3d TAC  = 7 mice, 3d Sham  = 7 mice, 6d TAC  = 6 mice, 6d Sham  = 4 mice, 21d TAC  = 10 mice, 21d Sham  = 10 mice. (H) Temporal development of leukocyte/immune cell ratio was determined for baseline animals as well as at 3, 6 and 21 days after TAC. Mean ±SEM; n PMN/macrophages: Baseline  = 8 mice, 3d TAC  = 5 mice, 6d TAC  = 6 mice, 21d TAC  = 5 mice; n DCs: Baseline  = 4 mice, 3d TAC  = 4 mice, 6d TAC  = 8 mice, 21d TAC  = 7 mice. (I) Flow cytometric quantification of monocytes in the blood. Mean ±SEM; n: Baseline  = 10 mice, 3d TAC  = 9 mice, 3d Sham  = 7 mice, 6d TAC  = 7 mice, 6d Sham  = 4 mice, 21d TAC  = 5 mice, 21d Sham  = 4 mice. *P<0.05, **P<0.01, ***P<0.001.

We studied the dynamics of phagocyte accumulation following cardiac hypertrophy using a murine model of TAC. Hereby, the determination of the Heart-Weight/Body-Weight-Index (HW/BW-Index) of the mice establishes the development of cardiac hypertrophy. The HW/BW-Index of TAC operated mice increased significantly on day 3 and 6 in comparison to sham controls and was still significantly elevated 3 weeks after TAC induction (Fig. 1A). The increased HW/BW-ratio is due to an increased heart weight because the body weight remained stable throughout the experimental duration (mean heart weight: 124.8 mg±12.85 mg/heart in healthy animals versus 145.9 mg±16.11 mg, 164.4 mg±27,38 and 170.8 mg±34.53 mg, 3, 6 and 21 days after TAC, respectively), indicating cardiac hypertrophy.

After preparation of the left ventricle (LV) and enzymatic digestion, the cardiac tissue suspension was stained for flow cytometry. To study the impact of immune cells in the process of cardiac remodeling, the single cell suspension was stained for the common leukocyte antigen CD45. Cardiac hypertrophy, provoked by TAC, significantly increased immune cell numbers in the LV tissue 3, 6 and 21 days following surgical intervention (Fig. 1B), indicating cardiac inflammation. To determine the composition of immune cells, leukocytes were distinguished via surface staining for F4/80 (tissue macrophages), Ly6C (macrophage subpopulations), CD11c (dendritic cells) and Gr1 or Ly6G (neutrophils; see gating strategy in Fig. 1C–E). A significant increase in macrophages in the cardiac tissue was observed 3 days after TAC, which peaked at day 6 (Fig. 1F), whereas neutrophils were significantly increased at day 3 (Fig 1G). At day 21 following TAC the number of macrophages and neutrophils in the heart were still elevated, although it did not reach the level of significance. Regarding the possible importance of leukocytes in the process of LV hypertrophy the proportion of macrophages, neutrophils and dendritic cells were examined in relation to all immune cells at the three time points and at baseline level. The phagocyte/immune cell ratio revealed that macrophages were the dominating cell population, representing more than half (mean: 53.55%) of the cardiac immune cells at day 6 following TAC, whereas the amount of neutrophils did not exceed 15% and the dendritic cells stayed below 5.4% and were not influenced by pressure overload (Fig. 1H). Moreover, whether or not a systemic reaction of the immune system to the stimulus of pressure overload occurred was examined. For this the number of monocytes in the circulation was ascertained at 3, 6 and 21 days after TAC (Fig. 1I). Although monocyte numbers appear to increase in response to surgical intervention, only the TAC-operated mice had significantly high numbers. Taken together these findings indicate that pressure overload led to cardiac inflammation as characterized by the accumulation of phagocytes.

### Macrophages during LV remodeling

**Figure 2:**
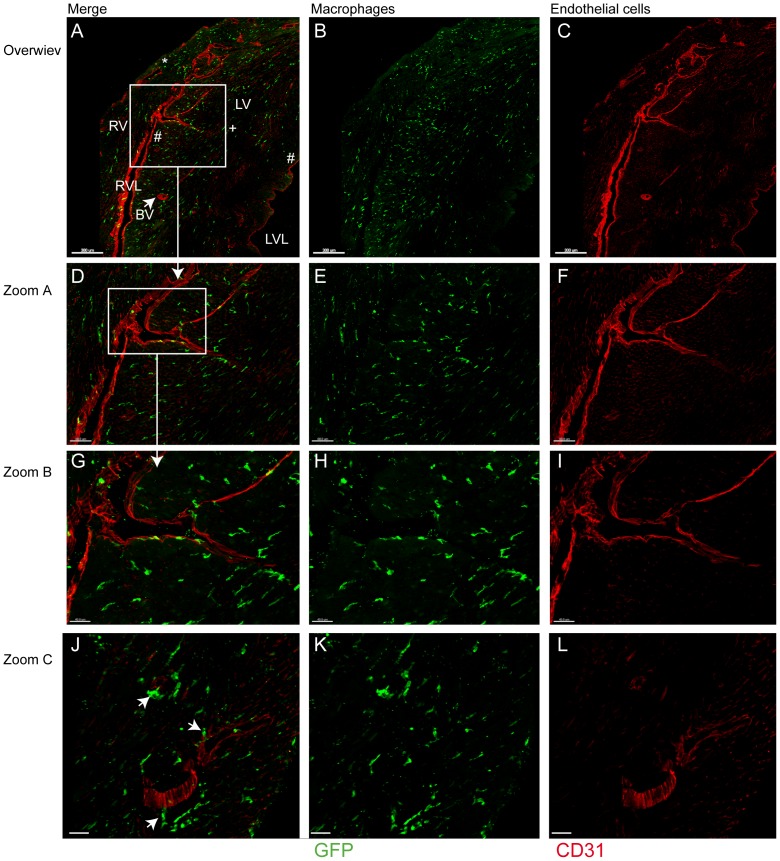
Macrophage localization in the heart determined by immunostaining. (A) Representative cardiac sections of CX3CR1^GFP/+^ mice 6 d after TAC, exhibiting macrophages in green, CD31 visualized in red by antibody staining. (A–C) Overwiew; * indicates the epicardial, + the mid-myocardial and # the endocardial regions; LV marks the left ventricle and RV the right ventricle. The arrow/BV marks one exemplary cardiac blood vessel. Scale bar: 300 µm. (D–F) Zoom in on the mid-myocardial and endocardial regions of the LV at the border to the RV. Scale bar: 80 µm; (G–I) Zoom in on the endocardial border zone of the LV and RV. Scale bar: 40 µm. (J–L) Zoom in on cardiac blood vessels in the mid-myocardial region of the LV, arrows indicate neighbouring macrophages. Scale bar: 35 µm.

For in situ localization of immune cells cryosections of cardiac tissue were examined. As macrophages proved to be the most common immune cells in the flow cytometry analysis, cryosections were prepared from hearts of CX3CR1^GFP/+^ transgenic mice. In these mice one allele of the CX3CR1 gene, the monocyte/macrophage-specific receptor for the membrane-tethered chemokine fractalkine has been replaced by the gene encoding enhanced green fluorescent protein (GFP) which facilitates monocyte/macrophage identification in cardiac tissue [30]. Thereby, cardiac macrophages were observed mainly in the endocardial and myocardial region of the RV lumen, whereas these immune cells were less abundant in the endocardial region of the LV lumen (Fig. 2 A–C). In intermediate fluorescence microscopical magnification of the endocardial and mid-myocardial regions of the RV lumen, the macrophages appeared to be evenly distributed (Fig. 2 D–F; 2G–I). A more detailed view revealed numerous macrophages in the vicinity of blood vessels (Fig. 2J–L).

**Figure 3:**
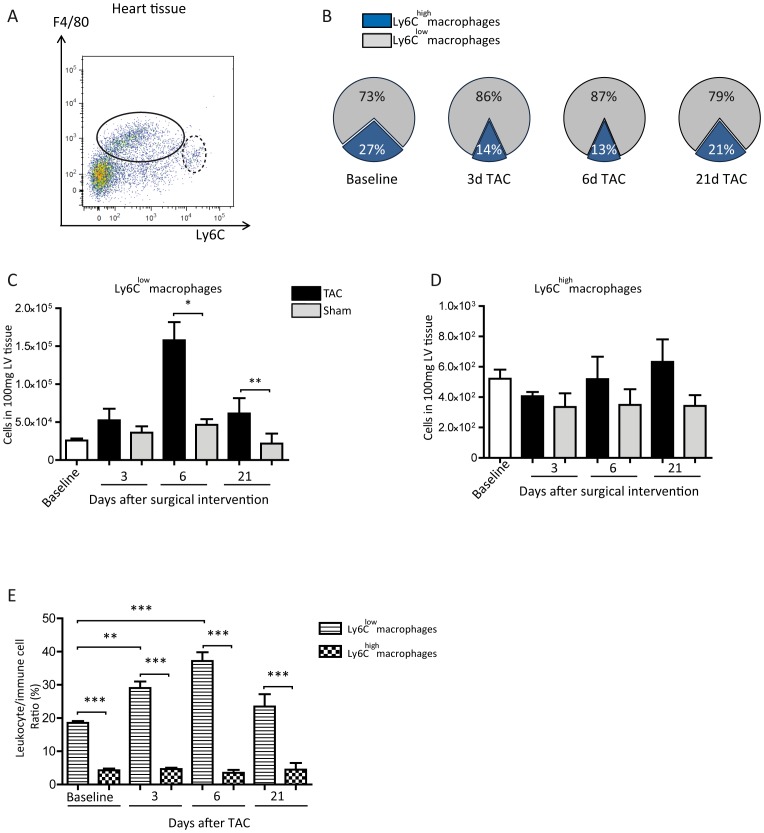
Cardiac phagocytes. (A) Gating strategy to determine the subpopulations of Ly6C^low^ (black ellipse) and Ly6C^high^ macrophages (black dashed ellipse) in the cardiac tissue via F4/80 and Ly6C staining, previously gating all immune cells; concatenated plots of 3 healthy baseline mice. (B) Pie graphs representing the relative proportion of Ly6C^low^ and Ly6C^high^ macrophages in relation to the whole population of cardiac macrophages. Graphs were drawn with Microsoft Excel 2010; results are shown as mean, n: Baseline  = 10 mice, 3d TAC  = 4 mice, 6d TAC  = 4 mice, 21d TAC  = 4 mice. (C, D) Flow cytometric quantification of Ly6C^high^ and Ly6C^low^ macrophages. (E) Flow cytometric quantification of the respective immune cell/macrophage ratio. (C, D, E) Mean ±SEM; n: Baseline  = 10 mice, 3d TAC  = 5 mice, 3d Sham  = 4 mice, 6d TAC  = 8 mice, 6d Sham  = 4 mice, 21d TAC  = 6 mice, 21d Sham  = 7 mice. *P<0.05, **P<0.01, ***P<0.001.

To further elucidate cardiac macrophages they were classified according to their expression pattern of Ly6C (see gating strategy in Fig. 3A). Thus, cardiac Ly6C^low^ and Ly6C^high^ macrophages were quantified by flow cytometry after TAC or sham operation. Ly6C^low^ macrophages represent the dominant part of the whole F4/80^+^ cardiac macrophage population in steady state and at all-time points after surgical intervention (Fig. 3B). Cardiac Ly6C^low^ macrophages increased after TAC reaching a maximum at day 6 and remained elevated until day 21, (Fig. 3C) while the number of Ly6C^high^ macrophages did not alter significantly at any time-point (Fig. 3D). To gain better insight into the proportion of both macrophage subpopulations, the numbers of Ly6C^low^ and Ly6C^high^ macrophages were normalized to the total immune cells. This evaluation validates the transient up-regulation of Ly6C^low^ macrophages due to pressure overload and emphasizes the large differences between the amounts of the two groups of macrophages (Fig. 3E). Taken together our findings indicate that after TAC the Ly6C^low^ macrophages form the major cardiac immune cell population with a temporal peak at day 6.

### Localization of phagocytes following TAC

**Figure 4:**
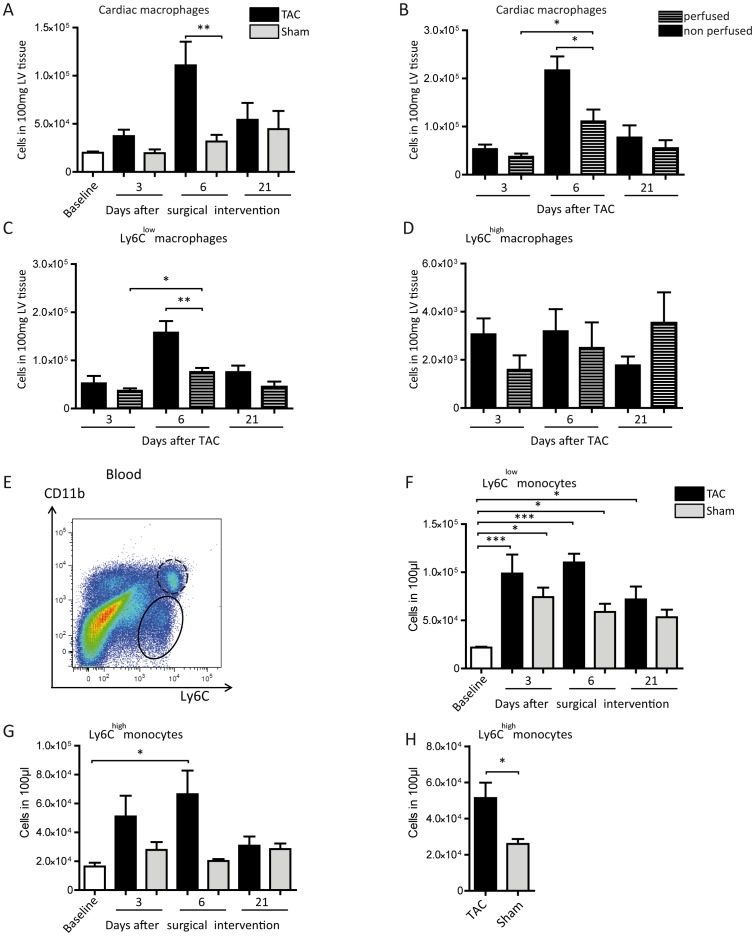
Flow cytometric quantification of macrophages in the heart and monocytes in the circulation. (A) Number of cardiac macrophages after PBS perfusion before explanting the hearts. Mean ±SEM; n: Baseline  = 10 mice, 3d TAC  = 6 mice, 3d Sham  = 4 mice, 6d TAC  = 8 mice, 6d Sham  = 4 mice, 21d TAC  = 6 mice, 21d Sham  = 5 mice. (B) Number of cardiac macrophages with and without PBS perfusion before explanting the hearts. Mean ±SEM; n of perfused animals: 3d TAC  = 6 mice, 6d TAC  = 7 mice, 21d TAC  = 6 mice; n of non-perfused animals: 3d TAC  = 6 mice, 6d TAC  = 5 mice, 21d TAC  = 6 mice. (C, D) Quantification of cardiac Ly6C^low^ (C) and Ly6C^high^ (D) macrophages with and without PBS perfusion before explanting the hearts. Mean ±SEM; n of perfused animals: 3d TAC  = 4 mice, 6d TAC  = 8 mice, 21d TAC  = 4 mice; n of non-perfused animals: 3d TAC  = 4 mice, 6d TAC  = 7 mice, 21d TAC  = 4 mice. (E) Gating strategy to determine the subpopulations of Ly6C^low^ (black ellipse) and Ly6C^high^ (blacked dashed ellipse) monocytes in the blood via CD11b and Ly6C staining, previously gating all single cells; concatenated plots of 3 healthy baseline mice. (F, G) Quantification of Ly6C^low^ (F) and Ly6C^high^ (G) monocytes in the blood. Mean ±SEM; n: Baseline  = 10 mice, 3d TAC  = 7 mice, 3d Sham  = 5 mice, 6d TAC  = 7 mice, 6d Sham  = 4 mice, 21d TAC  = 5 mice, 21d Sham  = 4 mice. (H) Accumulation of Ly6C^high^ monocyte numbers of all three time points after TAC- and sham-operation. Mean ±SEM; n: Baseline  = 10 mice, TAC  = 16 mice, sham  = 12 mice. *P<0.05, **P<0.01, ***P<0.001.

To exclude an influence of circulating immune cells on the analysis of intracardial macrophages, we perfused the circulation of the mice with PBS prior to explanting the hearts. In these mice, the increase of F4/80^+^ macrophages following TAC exhibited the same general picture (Fig. 4A) as without perfusion (Fig. 1F). However, the TAC-dependent accumulation of macrophages in the cardiac tissue reached the level of significance only at day 6. A direct comparison of cardiac macrophage numbers prepared from perfused versus non-perfused animals revealed a significant decline due to perfusion significant only at day 6 (Fig. 4B).

Interestingly, the number of cardiac Ly6C^low^ macrophages was reduced by perfusion of the circulation, whereas the lower frequency of Ly6C^high^ macrophages was not influenced by perfusion (Fig. 4C, D). This could be taken as a sign for a specific adhesion of Ly6C^low^ macrophages to the cardiac vessel walls in response to pressure overload. In accompanying blood samples Ly6C^low^ and Ly6C^high^ monocytes were ascertained according to the Ly6C surface expression (gating strategy in Fig. 4E). Analyzed during the development of LV hypertrophy, the Ly6C^low^ monocytes did not differ between TAC- and sham-operated groups, whereas TAC seemed to increase Ly6C^high^ monocyte numbers compared to sham-operated controls in the circulation (Fig. 4G, H). However, this monocyte population did not reach the level of significance in comparison with the respective sham groups. But, accumulatively the Ly6C^high^ cell numbers across all time-points revealed a significant increase of these cells in the blood due to pressure overload (Fig. 4I). These findings strengthen our hypothesis that the observed accumulation of Ly6C^low^ macrophages in the heart after TAC is a local phenomenon and most likely due to the cardiac immune response in the process of left ventricular hypertrophy.

### Expression of adhesion molecules

**Figure 5:**
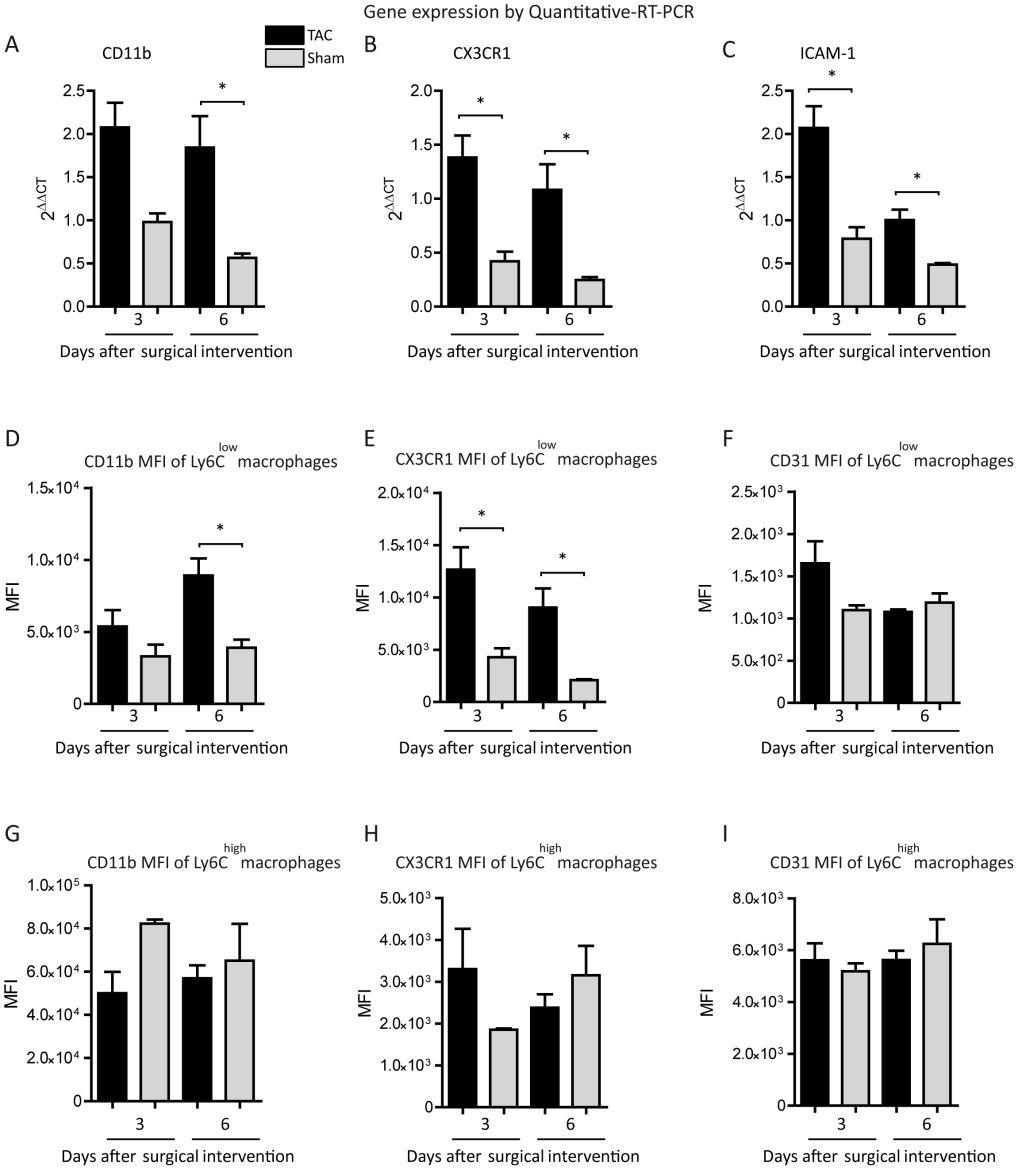
Analysis of adhesion molecules. (A–C) The mRNA-expression levels of adhesion molecules were quantified in the LV tissue of TAC- and sham- operated animals. The expression of the respective mRNA was normalized to healthy baseline controls. Mean ±SEM; n: 3d TAC  = 6 mice, 3d Sham  = 3 mice, 6d TAC  = 6 mice, 6d Sham  = 3 mice. (D–I) Flow cytometric delineation of the Mean Fluorescence Intensity (MFI) of CD11b (D, G), CX3CR1 (E, H) and CD31 (F, I) on the surface of cardiac Ly6C^low^ (E–G) and Ly6C^high^ (H–J) macrophages. Mean ±SEM; n: 3d TAC  = 5 mice, 3d Sham  = 3 mice, 6d TAC  = 8 mice, 6d Sham  = 3 mice. *P<0.05.

We hypothesized that the local attachment of Ly6C^low^ macrophages to the endothelium in response to pressure overload was due to up-regulation of adhesion molecules. Due to the accumulation of macrophages within the first week after TAC we focused on day 3 and 6. To test the above-mentioned hypothesis we analyzed whole LV tissue for expression of adhesion molecules by qRT-PCR. CD11b-, CX3CR1- and ICAM-1-mRNA expression were enhanced at day 3 and 6 after TAC (Fig. 5A–C), whereas CD31-mRNA expression was not altered compared to sham at the respective time points (data not shown).

To identify the responsible cell types for increased expression flow cytometry was applied and the mean fluorescence intensity (MFI) of the particular adhesion molecules was determined in conjunction with the particular cardiac cell population. On the surface of Ly6C^low^ macrophages from hypertrophied hearts MFI monitoring revealed a significant increase in CD11b fluorescence only at day 6 whereas CX3CR1 fluorescence was significantly elevated at both time points (Fig. 5D, E). Furthermore, CD31 fluorescence on Ly6C^low^ macrophages did not reach the level of significance at any time-point (Fig. 5F). In contrast, none of the determined antigens was altered on the surface of cardiac Ly6C^high^ macrophages in response to pressure overload (Fig. 5 G–I).

**Figure 6:**
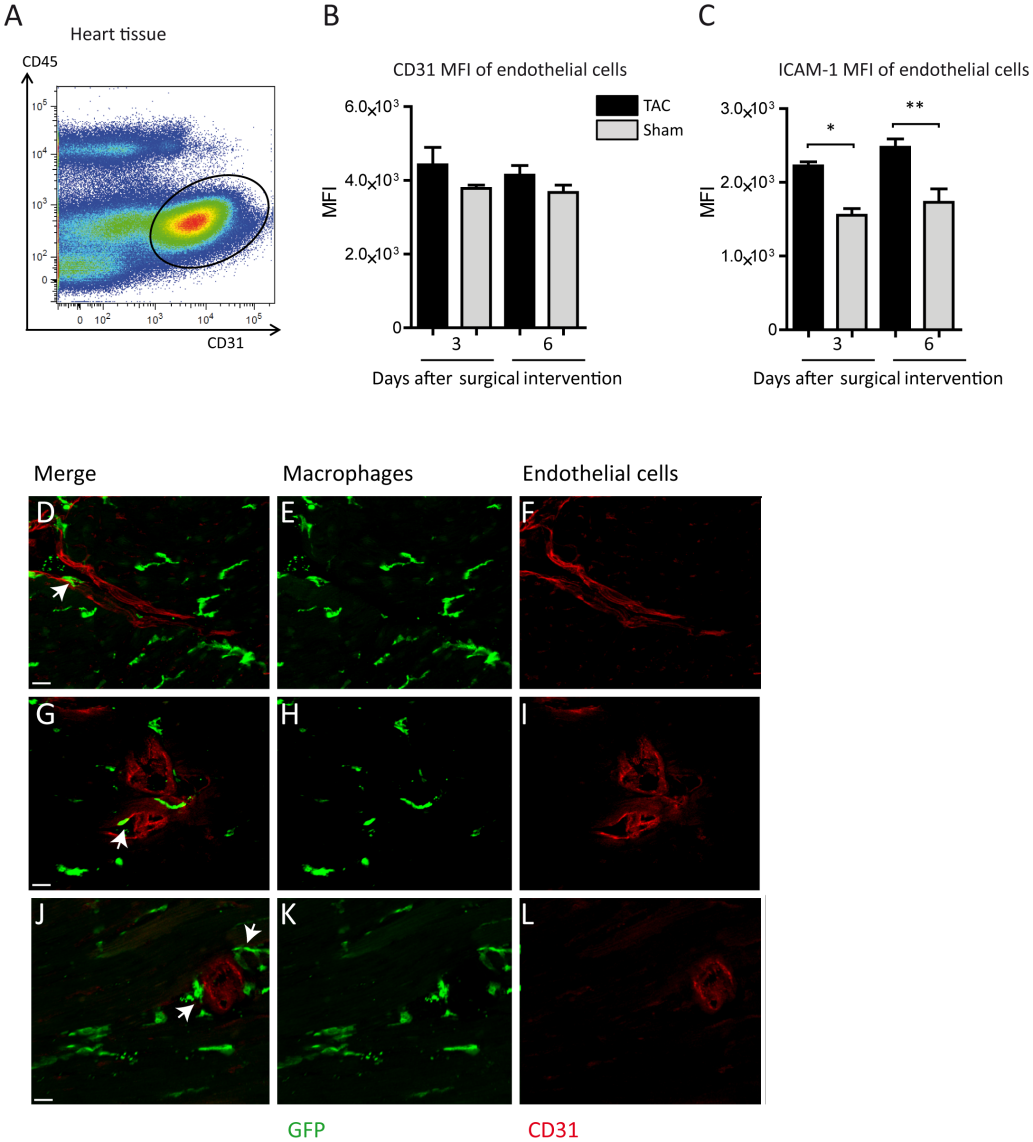
Macrophage adhesion in pressure overload. (A) Gating strategy to determine the endothelial cells among all single cells in the heart via CD45 and CD31 staining (black ellipse), previously gating all single cells; concatenated plots of 5 healthy baseline mice. (B, C) Flow cytometric delineation of CD31- and ICAM-1-MFI of cardiac endothelial cells. Mean ±SEM; n: 3d TAC  = 5 mice, 3d Sham  = 3 mice, 6d TAC  = 8 mice, 6d Sham  = 3 mice *P<0.05, **P<0.01. (D–L) Representative cardiac sections of CX3CR1GFP/+ mice 6 d after TAC. Monocytes/macrophages marked by GFP fluorescence in green, CD31 visualized in red by antibody staining. Arrows indicate monocytes/macrophages at different steps of recruitment: adhering to the intravascular endothelium (D–F), migrating through the endothelium (G–I) and directly neighboring the blood vessels (J–L). Scale bars: 30 µm (D–L).

Based on the finding, that mRNA expression levels of ICAM-1 are regulated in the first week following TAC, we analyzed the expression of this adhesion molecule on the surface of cardiac endothelial cells which were identified as CD45-negative and CD31-positive cells (see gating strategy in Fig. 6A) [31]. Here, the MFI of the macrophage adhesion molecule CD31 was slightly elevated at days 3 and 6 after TAC, while ICAM-1 reached the level of significance at days 3 and 6 (Fig. 6B, C). Taken together, the corresponding adhesion molecules ICAM-1 and CD11b were elevated due to pressure overload on endothelial cells and Ly6C^low^ macrophages. This may facilitate the adhesion of Ly6C^low^ macrophages to the cardiac vessel wall.

To further validate this conclusion monocytes and macrophages in the vicinity of cardiac blood vessels were visualised. Thereby the different steps of monocyte/macrophage recruitment to the cardiac tissue could be observed. GFP positive monocytes/macrophages attach to the vessel lumen migrate through the endothelium of small blood vessels and locate in close contact to the extravasal surface during pressure overload (Fig. 6D–L).

These findings highlight, that leukocyte adhesion happens in cooperation between immune cells and endothelium and that pressure overload provokes the expression of adhesion molecules, not only on macrophages, but also on endothelial cells.

## Discussion

Our findings highlight the dynamic changes of the cardiac immune response during the development of LV hypertrophy, which were characterized by significantly increased immune cell numbers within the first 21 days after TAC. The increase in CD45^+^ cells was due to elevated macrophage numbers at day 3 and 6 after TAC, as well as to a raised number of neutrophils at day 3. In this context, we demonstrated for the first time that LV pressure overload induced the accumulation of Ly6C^low^ macrophages in the myocardium. This selection of immune cells is in concordance with the specific expression and interaction between CD11b on the surface of monocytes/macrophages and ICAM1 on endothelial cells.

The amount of TAC-induced cardiac hypertrophy in this study amounted to about 7.5 HW/BW at day 6 and 21 which is in agreement with the current literature [11], [32]. Acute pressure overload is accompanied by a transient inflammatory response, which was defined by increased expression of pro-inflammatory cytokines in the cardiac tissue [33]. To gain further insight into the dynamics of this inflammatory process it is necessary to specify the involved immune cells. In previous studies it was shown that the number of MAC2^+^ macrophages in the heart increases during development of cardiac hypertrophy [34]. Therefore, in this study special interest was devoted to monocytes/macrophages. According to the current literature [8], [12], [35], macrophage populations in cardiac tissue were defined by flow cytometry as CD45^+^, F4/80^+^, Ly6C^high^ or Ly6C^low^. Tissue suspensions prepared from whole hearts for flow cytometry comprise immune cells from different sites (i.e., cells circulating in the blood stream or those residing in cardiac tissue and immune cells adhering to the border between the bloodstream and tissue). Previous studies, investigating cardiac tissue either analyzed perfused or non-perfused hearts [7], [32], [35], [36]. To differentiate between circulating, adhering and resident monocytes/macrophages we directly compared perfused and non-perfused hearts. Furthermore, we determined the amount of circulating monocytes in corresponding blood samples and evaluated the location of resident monocytes/macrophages by histology.

TAC induced a transient increase in F4/80^+^ monocytes/macrophages and Ly6G^+^ neutrophils. The dominant proportion of cardiac macrophages consisted of Ly6C^low^ cells in healthy baseline animals as well as in our model of cardiac hypertrophy. This cell population increased specifically in response to TAC, while Ly6C^high^ macrophages remained unaltered throughout the observation period. This was an unexpected finding as previous investigators reported that Ly6C^high^/M1 macrophages dominated the immune response at day 3 in response to myocardial ischemia [7], [9]. In case of MI Ly6C^high^/M1 macrophages exhibit phagocytic, proteolytic and inflammatory functions, whereas Ly6C^low^/M2 macrophages promote healing by myofibroblast accumulation, angiogenesis, and deposition of collagen [9]. However, MI leads to cellular necrosis ending in cellular degradation products inducing an acute severe inflammatory immune response within the first three days [7]. Attracted Ly6C^high^/M1 macrophages seem to play a central role in clearing this cellular debris [37], which does not emerge in comparable amounts during pressure overload. According to our results TAC seems to cause a more chronic stress to the heart, substantiated by a slow prolonged remodeling process which may be dominated by Ly6C^low^/M2 macrophages.

Perfusion of the circulation prior to explanting the heart reduced the number of cardiac macrophages, which continued to day 6 after TAC. In the perfused heart Ly6C^low^ monocytes/macrophages mirror the TAC-dependent temporal dynamics of the whole macrophage population determined in the cardiac tissue. Obviously the accumulation of macrophages in the heart appeared at a site which is accessible to the perfusion, indicating that these cells may be specifically attached to the endothelium. Attached cells could be demonstrated in histological preparations. It is important to mention that Ly6C^low^ monocytes/macrophages express more CX3CR1 than Ly6C^high^ cells. Therefore, the bright green cells in the histological sections can be referred to as Ly6C^low^ monocytes/macrophages [24], [25].

Remarkably, the Ly6C^high^ monocyte fraction in the blood was elevated in response to pressure overload, whereas their Ly6C^low^ counterpart did not vary significantly in this compartment. To further explain the specific accumulation of Ly6C^low^ macrophages in the heart, the expression of selected adhesion molecules was monitored in cardiac tissue. CD11b-, ICAM-1- and CX3CR1-mRNA expression proved to be elevated following pressure overload. To specify the adhesion molecules, they were quantified and assigned to certain cell-types via flow cytometry. We found up-regulation of CD11b and CX3CR1 expression on the surface of Ly6C^low^ macrophages within the first week after TAC, which was not detectable on the Ly6C^high^ macrophages. Simultaneously ICAM-1 the ligand of CD11b was elevated on cardiac endothelial cells as shown by MFI determination. This can facilitate the attachment of Ly6C^low^ monocytes to the vessel wall and may contribute to the increased number of these cells compared to the Ly6C^high^ monocytes/macrophages. Moreover, the increased expression of CX3CR1 on the surface of Ly6C^low^ monocytes may further promote their adhesion to the endothelium, although CX3CL1-mRNA expression in whole LV tissue was not influenced by TAC (results not shown). The specific up-regulation of the adhesion molecules has augmented the invasion of Ly6C^low^ monocytes/macrophages into the cardiac tissue during pressure overload. Interestingly, a CX3CR1 and CD11b dependent crawling of Ly6C^low^ monocytes at the luminal side of the endothelium has been recently described in the kidney [27], which may support our assumption. Although the increase in shear stress of the pre-stenotic vessels in our model can explain the up-regulation of ICAM-1 on the surface of endothelial cells, there is no obvious explanation for the changes on the surface of the Ly6C^low^ monocytes. Enhanced adhesion of monocytes from hypertensive patients has also been observed in an *in vitro* assay with human endothelial cells [38].

Moreover, our findings may contribute to understanding the role of Ly6C^low^ monocytes which have been described as specialized in surveying the endothelium [24], [25] and to require CX3CR1 and CD11b expression for this function [27]. We report that Ly6C^low^ monocytes/macrophages up-regulate these adhesion molecules and accumulate at the site of inflammation but do not extravasate extensively. Instead, a major part seems to be locally attached to the pre-stenotic endothelium of the cardiac vasculature in response to pressure overload. These cells contribute to tissue inflammation in this condition, and may represent a promising therapeutic target to prevent subsequent organ damage.

Although, many studies have been performed to characterize the specific roles of leukocytes in the case of remodeling after a cardiac ischemic injury, the role of these immune cells in cardiac remodeling during pressure overload is less defined. Here we have shown that the temporal succession of the immune response during the development of cardiac hypertrophy clearly differs from that after MI. Among the immune cells involved Ly6C^low^ monocytes/macrophages dominated the population at any time-point during the development of LV hypertrophy. In contrast, Ly6C^high^ monocytes/macrophages were the most abundant cells at day three after infarction, while Ly6C^low^ monocytes invaded the heart later. Furthermore, we demonstrated a simultaneous up-regulation of corresponding adhesion molecules on the surface of Ly6C^low^ monocytes/macrophages, as well as on endothelial cells, which may explain the selective enrichment of these cells in the cardiac tissue. According to our results the performance of Ly6C^low^ monocytes/macrophages is suitable for mediating the remodeling during the development of cardiac hypertrophy.

## References

[1] Koga K, Kenessey A, Ojamaa K (2013) Macrophage migration inhibitory factor antagonizes pressure overload-induced cardiac hypertrophy. Am J Physiol Heart Circ Physiol 304:H282–293. 23144312 10.1152/ajpheart.00595.2012

[2] Massie BM (2011) Novel targets for the treatment of heart failure: perspectives from a heart failure clinician and trialist. J Mol Cell Cardiol 51:438–440. 21549124 10.1016/j.yjmcc.2011.03.016

[3] Kurrelmeyer K, Kalra D, Bozkurt B, Wang F, Dibbs Z, et al (1998) Cardiac remodeling as a consequence and cause of progressive heart failure. Clin Cardiol 21:I14–19. 9853190 10.1002/clc.4960211304 PMC6656235

[4] Hunt SA, Abraham WT, Chin MH, Feldman AM, Francis GS, et al (2009) 2009 focused update incorporated into the ACC/AHA 2005 Guidelines for the Diagnosis and Management of Heart Failure in Adults: a report of the American College of Cardiology Foundation/American Heart Association Task Force on Practice Guidelines: developed in collaboration with the International Society for Heart and Lung Transplantation. Circulation 119:e391–479. 19324966 10.1161/CIRCULATIONAHA.109.192065

[5] Ehrentraut S, Lohner R, Schwederski M, Ehrentraut H, Boehm O, et al (2011) In vivo Toll-like receptor 4 antagonism restores cardiac function during endotoxemia. Shock 36:613–620. 22089127 10.1097/SHK.0b013e318235805f

[6] Tirziu D, Giordano FJ, Simons M (2010) Cell communications in the heart. Circulation 122:928–937. 20805439 10.1161/CIRCULATIONAHA.108.847731 PMC2941440

[7] Nahrendorf M, Swirski FK, Aikawa E, Stangenberg L, Wurdinger T, et al (2007) The healing myocardium sequentially mobilizes two monocyte subsets with divergent and complementary functions. The Journal of Exp Med 204:3037–3047. 18025128 10.1084/jem.20070885 PMC2118517

[8] Geissmann F, Gordon S, Hume DA, Mowat AM, Randolph GJ (2010) Unravelling mononuclear phagocyte heterogeneity. Nat Rev Immunol 10:453–460. 20467425 10.1038/nri2784 PMC3032581

[9] Nahrendorf M, Pittet MJ, Swirski FK (2010) Monocytes: protagonists of infarct inflammation and repair after myocardial infarction. Circulation 121:2437–2445. 20530020 10.1161/CIRCULATIONAHA.109.916346 PMC2892474

[10] Epelman S, Lavine KJ, Beaudin AE, Sojka DK, Carrero JA, et al (2014) Embryonic and adult-derived resident cardiac macrophages are maintained through distinct mechanisms at steady state and during inflammation. Immunity 40:91–104. 24439267 10.1016/j.immuni.2013.11.019 PMC3923301

[11] Velten M, Duerr GD, Pessies T, Schild J, Lohner R, et al (2012) Priming with synthetic oligonucleotides attenuates pressure overload-induced inflammation and cardiac hypertrophy in mice. Cardiovasc Res 96:422–432. 22977006 10.1093/cvr/cvs280

[12] Schiwon M, Weisheit C, Franken L, Gutweiler S, Dixit A, et al (2014) Crosstalk between Sentinel and Helper Macrophages Permits Neutrophil Migration into Infected Uroepithelium. Cell 156:456–468. 24485454 10.1016/j.cell.2014.01.006 PMC4258064

[13] Mollmann H, Nef HM, Troidl C (2010) ‘Turning the right screw’: targeting the interleukin-6 receptor to reduce unfavourable tissue remodelling after myocardial infarction. Cardiovasc Res 87:395–396. 20558440 10.1093/cvr/cvq186

[14] Roger VL (2007) Epidemiology of myocardial infarction. Med Clin North Am 91: 537–552; ix. 10.1016/j.mcna.2007.03.007 PMC2537993 17640535

[15] Panizzi P, Swirski FK, Figueiredo JL, Waterman P, Sosnovik DE, et al (2010) Impaired infarct healing in atherosclerotic mice with Ly-6C(hi) monocytosis. J Am Coll Cardiol 55:1629–1638. 20378083 10.1016/j.jacc.2009.08.089 PMC2852892

[16] Weber C, Erl W, Weber KS, Weber PC (1997) HMG-CoA reductase inhibitors decrease CD11b expression and CD11b-dependent adhesion of monocytes to endothelium and reduce increased adhesiveness of monocytes isolated from patients with hypercholesterolemia. J Am Coll Cardiol 30:1212–1217. 9350917 10.1016/s0735-1097(97)00324-0

[17] Herter J, Zarbock A (2013) Integrin Regulation during Leukocyte Recruitment. J Immunol 190:4451–4457. 23606722 10.4049/jimmunol.1203179

[18] Yang L, Froio RM, Sciuto TE, Dvorak AM, Alon R, et al (2005) ICAM-1 regulates neutrophil adhesion and transcellular migration of TNF-alpha-activated vascular endothelium under flow. Blood 106:584–592. 15811956 10.1182/blood-2004-12-4942 PMC1635241

[19] Kellermair J, Redwan B, Alias S, Jabkowski J, Panzenboeck A, et al (2013) Platelet endothelial cell adhesion molecule 1 deficiency misguides venous thrombus resolution. Blood 122:3376–3384. 24081660 10.1182/blood-2013-04-499558

[20] Muller WA (2003) Leukocyte-endothelial-cell interactions in leukocyte transmigration and the inflammatory response. Trends Immunol 24:327–334. 12810109 10.1016/s1471-4906(03)00117-0

[21] Imai T, Hieshima K, Haskell C, Baba M, Nagira M, et al (1997) Identification and molecular characterization of fractalkine receptor CX3CR1, which mediates both leukocyte migration and adhesion. Cell 91:521–530. 9390561 10.1016/s0092-8674(00)80438-9

[22] Auffray C, Fogg DK, Narni-Mancinelli E, Senechal B, Trouillet C, et al (2009) CX3CR1+ CD115+ CD135+ common macrophage/DC precursors and the role of CX3CR1 in their response to inflammation. J Exp Med 206:595–606. 19273628 10.1084/jem.20081385 PMC2699130

[23] Hochheiser K, Heuser C, Krause TA, Teteris S, Ilias A, et al (2013) Exclusive CX3CR1 dependence of kidney DCs impacts glomerulonephritis progression. J Clin Invest 123:4242–4254. 23999431 10.1172/JCI70143 PMC3784547

[24] Geissmann F, Jung S, Littman DR (2003) Blood monocytes consist of two principal subsets with distinct migratory properties. Immunity 19:71–82. 12871640 10.1016/s1074-7613(03)00174-2

[25] Geissmann F, Manz MG, Jung S, Sieweke MH, Merad M, et al (2010) Development of monocytes, macrophages, and dendritic cells. Science 327:656–661. 20133564 10.1126/science.1178331 PMC2887389

[26] Auffray C, Fogg D, Garfa M, Elain G, Join-Lambert O, et al (2007) Monitoring of blood vessels and tissues by a population of monocytes with patrolling behavior. Science 317:666–670. 17673663 10.1126/science.1142883

[27] Carlin LM, Stamatiades EG, Auffray C, Hanna RN, Glover L, et al (2013) Nr4a1-dependent Ly6C(low) monocytes monitor endothelial cells and orchestrate their disposal. Cell 153:362–375. 23582326 10.1016/j.cell.2013.03.010 PMC3898614

[28] Winer J, Jung CK, Shackel I, Williams PM (1999) Development and validation of real-time quantitative reverse transcriptase-polymerase chain reaction for monitoring gene expression in cardiac myocytes in vitro. Anal Biochem 270:41–49. 10328763 10.1006/abio.1999.4085

[29] Livak KJ, Schmittgen TD (2001) Analysis of relative gene expression data using real-time quantitative PCR and the 2(-Delta Delta C(T)) Method. Methods 25:402–408. 11846609 10.1006/meth.2001.1262

[30] Jung S, Aliberti J, Graemmel P, Sunshine MJ, Kreutzberg GW, et al (2000) Analysis of fractalkine receptor CX(3)CR1 function by targeted deletion and green fluorescent protein reporter gene insertion. Mol Cell Biol 20:4106–4114. 10805752 10.1128/mcb.20.11.4106-4114.2000 PMC85780

[31] Pratumvinit B, Reesukumal K, Janebodin K, Ieronimakis N, Reyes M (2013) Isolation, characterization, and transplantation of cardiac endothelial cells. Biomed Res Int 2013:359412. 24282814 10.1155/2013/359412 PMC3825130

[32] Souders CA, Borg TK, Banerjee I, Baudino TA (2012) Pressure overload induces early morphological changes in the heart. Am J Pathol 181:1226–1235. 22954422 10.1016/j.ajpath.2012.06.015 PMC3463627

[33] Baumgarten G, Knuefermann P, Kalra D, Gao F, Taffet GE, et al (2002) Load-dependent and -independent regulation of proinflammatory cytokine and cytokine receptor gene expression in the adult mammalian heart. Circulation 105:2192–2197. 11994254 10.1161/01.cir.0000015608.37608.18

[34] Velten M, Duerr GD, Pessies T, Schild J, Lohner R, et al (2012) Priming with synthetic oligonucleotides attenuates pressure overload-induced inflammation and cardiac hypertrophy in mice. Cardiovasc Res 96:422–432. 22977006 10.1093/cvr/cvs280

[35] Pinto AR, Paolicelli R, Salimova E, Gospocic J, Slonimsky E, et al (2012) An abundant tissue macrophage population in the adult murine heart with a distinct alternatively-activated macrophage profile. PLoS One 7:e36814. 22590615 10.1371/journal.pone.0036814 PMC3349649

[36] Yan X, Anzai A, Katsumata Y, Matsuhashi T, Ito K, et al (2013) Temporal dynamics of cardiac immune cell accumulation following acute myocardial infarction. J Mol Cell Cardiol 62:24–35. 23644221 10.1016/j.yjmcc.2013.04.023

[37] Frantz S, Nahrendorf M (2014) Cardiac macrophages and their role in ischaemic heart disease. Cardiovasc Res 102:240–248. 24501331 10.1093/cvr/cvu025 PMC3989449

[38] Syrbe U, Moebes A, Scholze J, Swidsinski A, Dorffel Y (2007) Effects of the angiotensin II type 1 receptor antagonist telmisartan on monocyte adhesion and activation in patients with essential hypertension. Hypertens Res 30:521–528. 17664855 10.1291/hypres.30.521

